# Prophenoloxidase of *Odontotermes formosanus* (Shiraki) (Blattodea: Termitidae) Is a Key Gene in Melanization and Has a Defensive Role during Bacterial Infection

**DOI:** 10.3390/ijms24010406

**Published:** 2022-12-26

**Authors:** Zhiqiang Wang, Jian Luo, Kai Feng, Yujingyun Zhou, Fang Tang

**Affiliations:** 1 Co-Innovation Center for Sustainable Forestry in Southern China, Nanjing Forestry University, Nanjing 210037, China; 2 College of Forestry, Nanjing Forestry University, Nanjing 210037, China

**Keywords:** *Odontotermes formosanus* (Shiraki), Prophenoloxidase, *Serratia marcescens* Bizio, *Bacillus thuringiensis*, RNA interference

## Abstract

Melanization mediated by the prophenoloxidase (PPO)-activating system is an important innate immunity to fight pathogens in insects. In this study, the in vitro time-dependent increase in the intensity of melanization and phenoloxidase (PO) activity from the hemolymph of *Odontotermes formosanus* (Shiraki) challenged by pathogenic bacteria was detected. PPO is one of the key genes in melanization pathway, whereas the molecular characteristics and functions of *O. formosanus* PPO are unclear. The *OfPPO* gene was cloned and characterized. The open reading frame of *OfPPO* is 2085 bp in length and encodes a 79.497 kDa protein with 694 amino acids. A BLASTx search and phylogenetic analyses revealed that OfPPO shares a high degree of homology to the Blattodea PPOs. Moreover, real-time fluorescent quantitative PCR analysis showed that *OfPPO* is ubiquitously expressed in all castes and tissues examined, with the highest expression in workers and variable expression patterns in tissues of different termite castes. Furthermore, the expression of *OfPPO* was significantly induced in *O. formosanus* infected by pathogenic bacteria. Intriguingly, in combination with silencing of *OfPPO* expression, pathogenic bacteria challenge caused greatly increased mortality of *O. formosanus*. These results suggest that *OfPPO* plays a role in defense against bacteria and highlight the novel termite control strategy combining pathogenic bacteria application with termite PPO silencing.

## 1. Introduction

Termites are important agricultural pests that can damage to nearly anything made of cellulose, leading to an annual loss of 40 billion dollars worldwide [1,2]. They are eusocial insects with definite castes, such as king, queen, soldiers, and workers, within colonies. The *Odontotermes formosanus* (Shiraki) (Blattodea: Termitidae) is a notorious termite species that is distributed in China, India, Japan, Myanmar, Vietnam, and Thailand [3,4]. *O. formosanus* not only damages crops, plantations, and forests but is also a major concern for disasters if they build numerous hidden chambers in dams, leading to structural instability [5,6]. *O. formosanus* populations can be controlled by applications of pesticides, but the excessive use of pesticides can seriously affect the human health and environment safety [7]. Therefore, there is an urgent need to develop environmentally friendly biological control. *Serratia marcescens* and *Bacillus thuringiensis* have promising applications in the biological control of termites [8,9,10,11,12]. *S. marcescens*, a pathogenic bacterium recently isolated from dead *O. formosanus*, has a pathogenic effect on *O. formosanus* [13]. Laboratory studies have demonstrated that *B. thuringiensis*, the mostly and widely used bacterial insecticide, has a good control effect on termite [11,12]. Thus, these two insect pathogens have great potential to be good microbial control pathogens against *O. formosanus*.

Insects combat infection by pathogens through mounting powerful immune responses (cellular and humoral immunity), which reduce the virulence of pathogens, such as apoptosis, phagocytosis, autophagy, melanization, the JAK/STAT pathway, the Toll pathway and the immune deficiency (Imd) pathway, etc. [14,15,16,17]. Currently, immune genes are regarded as potential target genes for RNA interference (RNAi)-based control strategies. The RNAi technique has potential practical value for the development of new tools for the management of insect pests [18,19]. For example, the Imd pathway is involved in the antifungal response of *Anthonomus grandis* and interference with the relish gene by RNAi can increase the susceptibility of *A. grandis* to *Metarhizium anisopliae* [20]. Inhibiting apoptosis related genes of Colorado potato beetle is an effective RNAi-mediated control measure [21]. The silencing of the locust apoptosis-related gene can reduce insect immunity and increase insect susceptibility to fungal pathogens [22]. The knockdown of several genes in the Toll pathway also can increase pest lethality, which provides new targets for pest control [23].

In insects, melanization reaction is one of the most important immune processes. Mounting evidence suggests that the antimicrobial effect of melanization is an indispensable component of the insect defense mechanism to protect the insects against microbes [24,25]. Phenoloxidase (PO) catalyzes the conversion of phenols to quinones via an oxidation reaction and promotes the formation of melanin which directly encapsulates and clears away foreign pathogens [26,27,28]. Active PO is formed by prophenoloxidase (PPO) in a specific protease system [29,30]. Previous research suggested that the PPO-mediated melanization pathway was indispensable for insects to defend against pathogens, in which PPO played a critical role in regulating immune responses [31]. According to the information mentioned above, interfering with melanization pathway by RNAi technology can be an effective method to control termites. The potentially critical target gene is PPO in the RNAi-mediated control strategy. However, its characteristics and function have not been defined yet.

In this study, we used pathogenic bacteria and *O. formosanus* as an infection model to investigate the effects of pathogenic bacterial infection on the hemolymph melanization of *O. formosanus*. We cloned *OfPPO* gene and analyzed the function of *OfPPO* in *O. formosanus*. *OfPPO* was determined to serve as an important component of hemolymph melanization and to defend insects against pathogenic bacteria. This study not only characterizes the function of PPO gene of *O. formosanus*, but also provides insights for the integrated control of *O. formosanus* with RNAi and pathogenic bacteria mediated control strategies.

## 2. Results

### 2.1. Melanization Accelerated and PO Activity Induced by SM1 and Bt in O. formosanus

**Figure 1:**
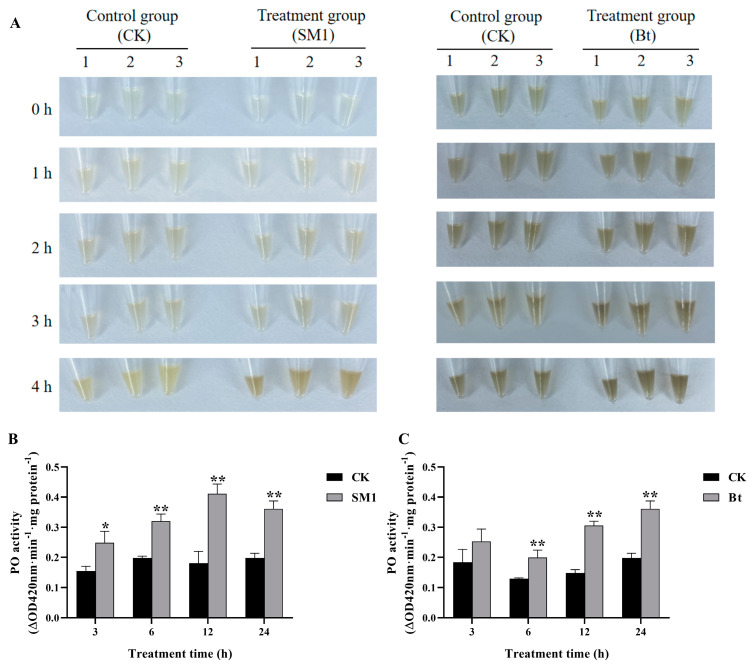
The melanization and enzymatic activity of PO in *O. formosanus* treated by SM1 and Bt. (**A**): The level of melanization in the hemolymph of *O. formosanus* infected with bacteria treated for 0, 1, 2, 3 and 4 h. The treatment groups were treated with SM1 or Bt. The control group was treated with culture medium (CK). The melanization reaction was recorded by photography at different time points from 0 to 4 h. (**B**): PO activity in *O. formosanus* infected by SM1. The control group was treated with culture medium (CK). (**C**): PO activity in *O. formosanus* infected by Bt. The control group was treated with culture medium (CK). The data are presented as the mean ± SD of three replicates. The asterisks indicate significant differences between the treatments groups and the control groups (* *p* < 0.05, ** *p* < 0.01).

Melanization of hemolymph from *O. formosanus* infected with SM1 or Bt was investigated. As shown in Figure 1A, compared to the uninfected groups, accelerated melanization was observed in *O. formosanus* treated by SM1 and Bt for 0, 1, 2, 3, and 4 h. Moreover, the elevated PO activity occurred in the bacteria-infected groups at most of the time points post treatment (Figure 1B,C).

### 2.2. Cloning and Characterization of OfPPO in O. formosanus

**Figure 2:**
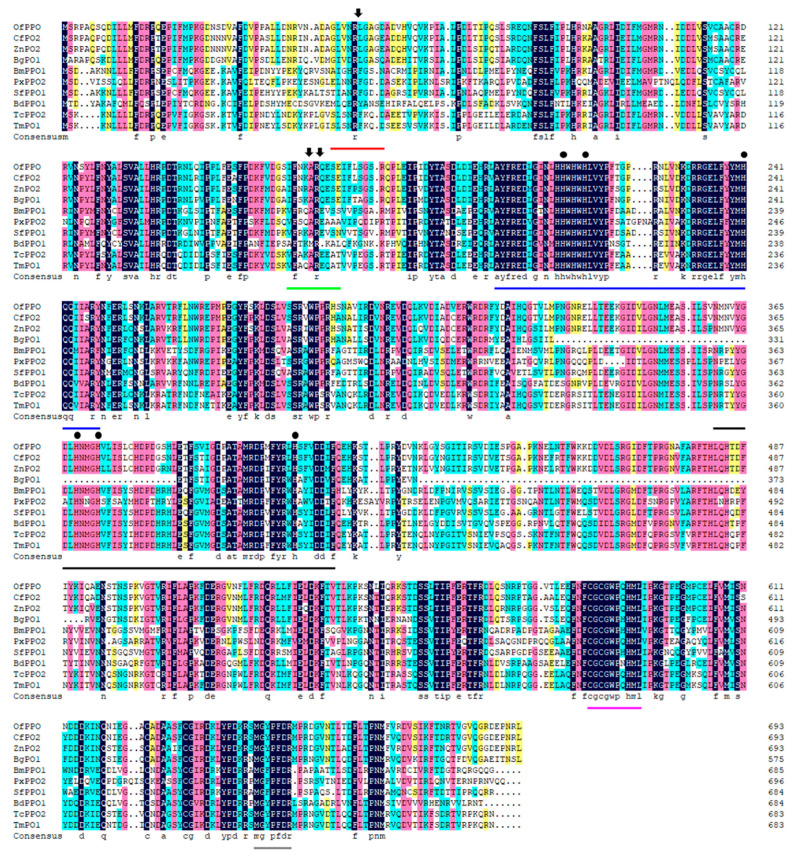
Multiple amino acid sequence alignment of insect PPOs. Protein sequences of from *Odontotermes formosanus* PPO (OfPPO), *Coptotermes formosanus* PO2 (CfPO2, AHB39936.1), *Zootermopsis nevadensis* PO2 (ZnPO2, XP_021921811.1), *Blattella germanica* PO1 (BgPO1, PSN38098.1), *Bombyx mori* PPO1 (BmPPO1, AAG09304), *Plutella xyostella* PPO2 (PxPPO2, ACS36209.1), *Spodoptera frugiperda* PPO1 (SfPPO1, ABB92834), *Bactrocera dorsalis* PPO1 (BdPPO1, AFP81887), *Tribolium castaneum* PPO2 (TcPPO2, NP_001034522), and *Tenebrio madens* PO1 (TmPO1, XP_044260894.1) were aligned using DNAMAN. The two potential proteolytic cleavage sites were shown by red and green lines, and the predicted cleavage bonds were marked with black arrows. CuA and CuB were underlined with blue and black lines respectively, and the conserved six histidine residues are marked with black dots. The thiol ester sites and C-terminal conserved motif were underlined with pink and gray lines, respectively.

**Figure 3:**
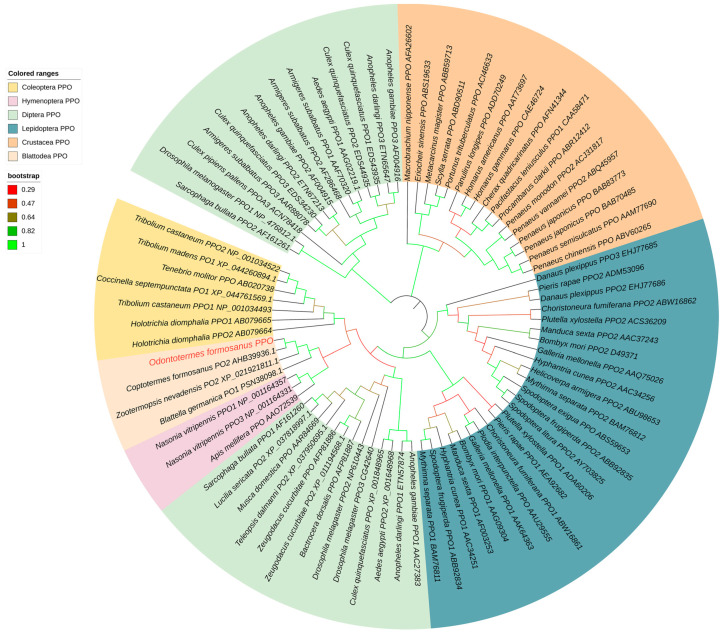
Phylogenetic analysis of the protein sequences of 82 PPOs, including 38 insects and 16 crustaceans. The PPO gene name was shown as the Latin name of the species, and the NCBI gene accession number was added. The *O. formosanus* PPO was marked in red. The branches specific for Lepidoptera, Blattodea, Coleoptera, Hymenoptera, Crustacea, and Diptera PPOs were shaded in different colors. Branch colors represent bootstrap values.

We successfully cloned the complete cDNA of the *OfPPO* gene (accession number: OP828923) and found that *OfPPO* contains an ORF of 2085 bp encoding a 694 amino acid protein with a molecular weight of 79.497 kDa and a predicted isoelectric point (pI) of 6.01. Alignment of amino acid sequences of OfPPO with the other nine insect PPOs showed that six motifs were extremely well-conserved in OfPPO (Figure 2). Among the six motifs, two were annotated as potential proteolytic cleavage sites, another two were copper binding sites (CuA and CuB), one was a thiol-ester-like motif, and the other was a conserved C-terminal motif. Comparing the amino acid sequence using BLASTx, the OfPPO protein exhibited high degree of identity ranged between 59.68% and 93% with the PPO of other insect species (Table 1). The OfPPO protein sequence is highly similar to insect PPO sequences of the *Blattella*. OfPPO shares 92.8% pairwise identity with *Coptotermes formosanus* PO2 (CfPO2) at the amino acid level and 87.9% and 79.34% pairwise identity with *Zootermopsis nevadensis* PO2 (ZnPO2) and *Blattella germanica* PO1 (BgPO1), respectively (Table 1). Based on the deduced OfPPO amino acid sequence, 81 invertebrate PPO (PO) sequences were selected, and a high-accuracy phylogenetic tree was generated using MEGA X. The phylogenetic tree was divided into six different groups. In particular, OfPPO was most clustered with Blattodea PPO (Figure 3). This result showed that OfPPO was highly similar to the PPO of Blattodea.

### 2.3. Caste- and Tissue-Specific Expression Profiles of OfPPO in O. formosanus

**Figure 4:**
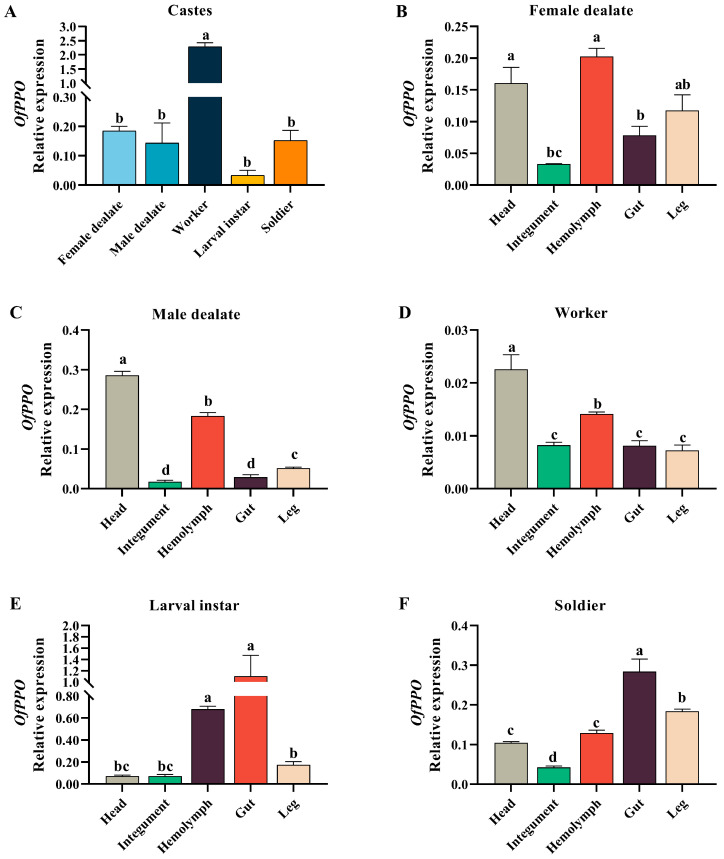
Expression profiles of *OfPPO* in different castes and tissues of *O. formosanus*. (**A**) *OfPPO* mRNA expression levels in five castes. (**B**) OfPPO mRNA expression levels in five tissues of female dealates. (**C**) OfPPO mRNA expression levels in five tissues of male dealates. (**D**) *OfPPO* mRNA expression levels in five tissues of worker. (**E**) *OfPPO* mRNA expression levels in five tissues of larval instars. (**F**) *OfPPO* mRNA expression levels in five tissues of soldiers. The data are presented as the mean ± SD of three replicate samples. The same letters on the error bars indicate no significant difference in *OfPPO* expression (*p* > 0.05).

The mRNA levels of *OfPPO* from different castes of *O. formosanus* were analyzed using real-time fluorescent quantitative PCR (qPCR). The expression of *OfPPO* in the workers was significantly higher than that in soldiers, larval instars, and dealates (Figure 4A).

The expression of *OfPPO* was examined in different body parts as well. For female dealates, *OfPPO* was expressed at relatively high levels in the hemolymph and head (Figure 4B). For male dealates, *OfPPO* was expressed at the highest level in the head followed by the hemolymph (Figure 4C). For workers, the expression pattern of *OfPPO* was similar to that of male dealates (Figure 4D). For larval instars, *OfPPO* was expressed at high levels in the hemolymph and gut (Figure 4E). For soldiers, *OfPPO* was expressed at the highest level in the gut, followed by the leg (Figure 4F).

### 2.4. The OfPPO Expression Induced by SM1 and Bt in O. formosanus

**Figure 5:**
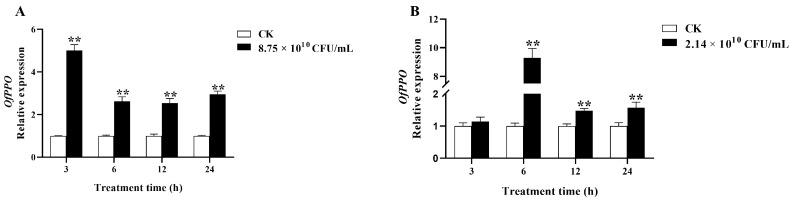
Elevated *OfPPO* expression in *O. formosanus* workers infected by SM1 (**A**) and Bt (**B**). The data are presented as the mean ± SD of three replicate samples. The asterisks indicate significant differences between the treatment groups and the control groups (** *p* < 0.01).

Next, we investigated whether *OfPPO* is involved in defense against bacterial infection. We measured the mRNA levels of *OfPPO* in *O. formosanus* challenged by SM1 and Bt, respectively. As shown in Figure 5A, the *OfPPO* expression was significantly increased after SM1 infection at all treated time points. Moreover, the transcript levels of *OfPPO* were significantly upregulated when treated by Bt for 6, 12, and 24 h, respectively (Figure 5B). In total, the results showed that SM1 and Bt challenges could induce the expression of *OfPPO* in *O. formosanus*.

### 2.5. The Function of OfPPO in Resisting SM1 and Bt Infection in O. formosanus

**Figure 6:**
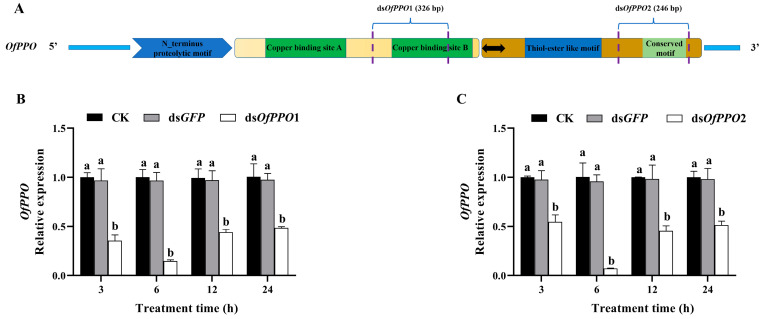
Analysis of the silencing efficiency of *OfPPO* in *O. formosanus*. (**A**) Conceptual diagrams illustrating the interference regions for designing dsRNA fragments (including ds*OfPPO*1 and ds*OfPPO*2). Black arrows indicate the regions for detecting the expression level of *OfPPO* by qPCR. (**B**) Relative expression levels of *OfPPO* in *O. formosanus* fed on ds*OfPPO*1. (**C**) Relative expression levels of *OfPPO* in *O. formosanus* fed on ds*OfPPO*2. The data are presented as the mean ± SD of three replicates. The same letters indicate no significant difference in the *OfPPO* expression (*p* > 0.05).

To evaluate the effectiveness of two dsRNAs from two important *OfPPO* domains, two in vitro synthesized dsRNAs (ds*OfPPO*1 and ds*OfPPO*2) were designed (Figure 6A). We found that ds*OfPPO*1 and ds*OfPPO*2 suppressed the transcript levels of *OfPPO* by 92.7% and 85.1% at 6 h, respectively (Figure 6B,C).

**Figure 7:**
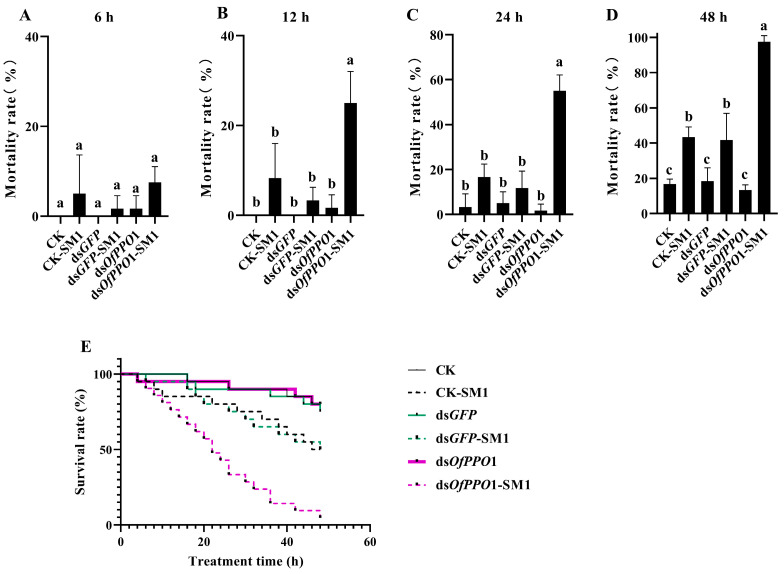
Bioassay test of SM1-challenged *O. formosanus* treated with ds*OfPPO*1. (**A**) The mortality rate of *O. formosanus* in each group at 6 h. (**B**) The mortality rate of *O. formosanus* in each group at 12 h. (**C**) The mortality rate of *O. formosanus* in each group at 24 h. (**D**) The mortality rate of *O. formosanus* in each group at 48 h. (**E**) The survival rate of SM1-challenged *O. formosanus* treated with ds*OfPPO*1. The data are presented as the mean ± SD of three replicates. In these figures, the same letters indicate no significant differences in the mortality of *O. formosanus* (*p* > 0.05).

**Figure 8:**
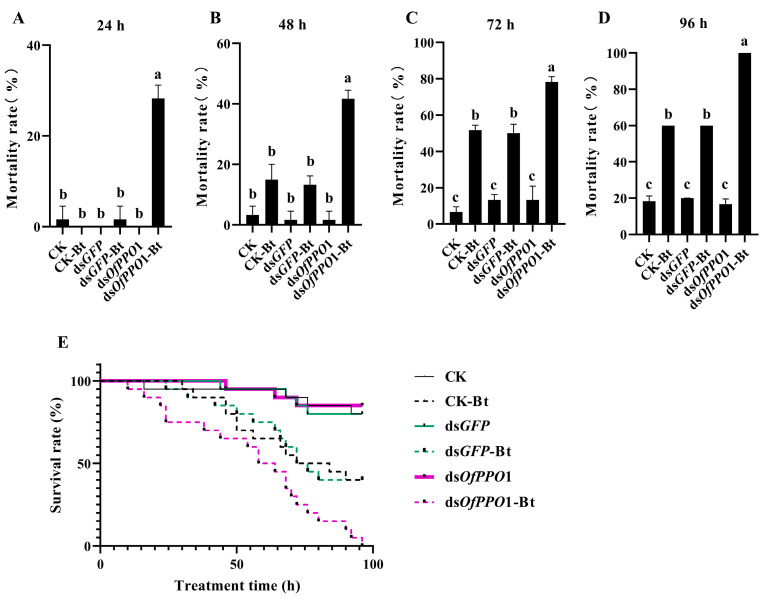
Bioassay test of Bt-challenged *O. formosanus* treated with ds*OfPPO*1. (**A**) The mortality rate of *O. formosanus* in each group at 24 h. (**B**) The mortality rate of *O. formosanus* in each group at 48 h. (**C**) The mortality rate of *O. formosanus* in each group at 72 h. (**D**) The mortality rate of *O. formosanus* in each group at 96 h. (**E**) The survival rate of Bt-challenged *O. formosanus* treated with ds*OfPPO*1. The data are presented as the mean ± SD of three replicates. In these figures, the same letters indicate no significant differences in the mortality of *O. formosanus* (*p* > 0.05).

The effect of *OfPPO* on bacterial infection was examined in vivo. After feeding *O. formosanus* for 6 h with sterilized water (negative control), *GFP* dsRNA, and ds*OfPPO*1 or ds*OfPPO*2, *O. formosanus* was exposed to SM1. The mortality of *O. formosanus* treated with ds*OfPPO*1 and SM1 was significantly increased compared with that of *O. formosanus* treated with ds*GFP* and SM1 at 12 and 24 h (Figure 7B,C). Interestingly, the mortality of *O. formosanus* treated with ds*OfPPO*1 and SM1 was almost 100% at 48 h (Figure 7D). In addition, the mortality of *O. formosanus* treated with ds*OfPPO*1 and Bt was significantly increased at 24, 48, and 72 h compared with that of *O. formosanus* treated with ds*GFP* and Bt (Figure 8A–C). Furthermore, the mortality of *O. formosanus* treated with ds*OfPPO*1 and Bt was almost 100% at 96 h (Figure 8D). Similar results were obtained when *O. formosanus* was treated with ds*OfPPO*2 and SM1 or ds*OfPPO*2 and Bt (Figures S1 and S2).

## 3. Discussion

The development of entomopathogens as classical, safe, eco-friendly, and sustainable biological control agents has achieved many successes in recent years [32]. Microbial control techniques involving entomopathogens have been developed as a nonchemical alternative for pest control [33]. Meanwhile, entomopathogens have received attention as sustainable environmentally friendly control agents for the control of the insects [8,9,12]. To combat pathogen infection, insects have evolved potent immune defense mechanisms, including cellular immunity and humoral immunity. In insect immunity, melanization is an important part of humoral immunity. It is well known that the melanization reaction is an important anti-pathogen strategy that attacks pathogenic bacteria in insects, thus reducing the damage caused by microorganisms to insects [29,34,35,36]. Growing evidence suggests that different insects have developed PPO-mediated melanization pathways to defend against infection [35,37,38]. In this study, we observed that melanization in *O. formosanus* was accelerated by SM1 and Bt infection. It demonstrated that termite melanization is activated in response to biocontrol bacterial infection, which is one of the major obstacles to the use of pathogenic bacteria for termite control. Thus, suppressing the melanization of termites can improve the effectiveness of biocontrol pathogen-mediated pest control.

PPO, as a zymogen, is an important component in the melanization pathway, which produces PO through a specific protease cascade. PO plays an important role in invertebrates, including wound healing [39], hemolymph clotting [40], and encapsulation or melanization of foreign pathogens [17,27]. In *Aedes aegypti*, the expression of PPOs could be induced after pathogen infection, and the melanization pathway was activated [41,42]. In *Spodoptera exigua* larval hemolymph, the expression of PPO was significantly induced, and melanin formation occurred after *Heliothis virescens ascovirus* infection [43]. The pea aphid defended against bacterial and fungal infection through upregulating the expression of PPO and improving the PO activity [30]. PO activity was prominent in the gut of *Reticulitermes flavipes*, which was useful for maintaining the digestive balance between the insect and the symbiont bacteria [44]. Due to the higher pathogenic pressure in the soil environment, the PO activity of *Nasutitermes acajutlae* was higher compared to arboreal termites [45]. In this study, we found that the expression of *OfPPO* and the activity of PO in *O. formosanus* infected by pathogenic bacteria gradually increased, suggesting that the PPO-mediated melanization has a conserved role in the defense against pathogens in insects.

Very little is known about the characteristics of PPO in *O. formosanus*. In this research, we first obtained a 2085 bp full-length DNA sequence of *OfPPO*, which contained specific conserved motifs (proteolytic cleavage sites, conserved copper binding sites, thiol ester-like motifs and C-terminal conserved motifs), which are regarded as expressed sequence tags for PPOs [46,47]. Therefore, we initially speculate that OfPPO has the same function as other insect PPOs, e.g., antibacterial function. In addition, the results of phylogenetic analysis of eighty-one PPO proteins indicated that OfPPO shares a common ancestor with *C. formosanus*, *Z. nevadensis*, and *B. germanica*, as these species cluster on adjacent branches. The results of gene structure and phylogenetic analysis indicated that OfPPO was relatively conserved among different invertebrates. In *Apis mellifera*, the expression levels of PPO in older pupae and adults were higher than those in larvae and younger pupae [47]. In *Hyphantria cunea*, the expression of PPO was detected in most stages of development [48]. In *Anopheles gambiae*, six PPO genes were expressed in different life stages [49]. Here, *OfPPO* was differentially expressed with respect to castes. Interestingly, workers showed the highest *OfPPO* expression in all castes. We hypothesized that workers often foraged outside of their colony and suffered diverse stresses, and thus had a higher immunity than soldiers and dealates. With respect to tissue expression, melanization mainly occurred in insect hemolymph, and the expression of *OfPPO* was relatively high in the hemolymph, which was consistent with a previous report [50,51].

RNAi is a promising new sustainable strategy for ecologically friendly pest control [52]. This technology achieves the inhibition of specific endogenous gene expression by double-stranded RNA (dsRNA) sequences. The effectiveness of RNAi has been proven in many species [52,53]. For example, immune function of *Spodoptera littoralis* larvae was destroyed by dsRNA molecules, which led to an increase in its susceptibility to Bt [54]. The regulatory genes in the Toll pathway were identified as new lethal targets for pest control [23]. Inhibitors of apoptosis were found to be potent target genes for RNAi-based control of pests [21,22]. In our study, we chose the *OfPPO* gene, the essential immune gene in melanization, as an RNAi target gene in *O. formosanus*. When *O. formosanus* treated by ds*OfPPO*s was inoculated with SM1 or Bt, the lethality of the pathogenic bacteria to *O. formosanus* was significantly increased. These results demonstrated that the PPO gene is a good candidate RNAi target for controlling *O. formosanus*. Some RNAi delivery systems have been developed to control pests, including the use of bacterial, viral, and fungal agents and genetically modified plants or the use of nanoparticles, as well as direct spraying or feeding of dsRNA [22,55]. *OfPPO* may control *O. formosanus* via these dsRNA delivery systems.

In summary, PPOs serve important roles in the resistance of insects to pathogenic bacterial infection. Our results defined the *OfPPO* expression distribution in castes and tissues. RNAi-mediated silencing of *OfPPO* demonstrated that knockdown of *OfPPO* expression facilitated pathogenic bacterial infection in *O. formosanus*. Therefore, PPO genes involved in the melanization reaction can be used as candidate targets for RNAi control.

## 4. Materials and Methods

### 4.1. Insects and Bacteria

*Odontotermes formosanus* colonies were obtained from Nanjing Forestry University (Nanjing, China) and kept in a dark environment at 25 ± 1 °C with 75% relative humidity (RH) that was maintained in a feeding device consisted of colony and foraging areas. The larval instars and soldiers from the colonies were utilized for caste- and tissue-specific gene expression analysis. The naïve female and male dealates were collected for caste- and tissue-specific gene expression analysis. The *Serratia marcescens* (SM1) and the *B. thuringiensis* (Bt) strains were stored at −80 °C in our laboratory. For experiments, SM1 was cultured in fermentation medium for 30 h in a shaker incubator with a temperature set at 28 ℃ and a rotation set at 180 rpm. Bt was cultured with lysogen broth under the same conditions for 6 days.

### 4.2. Melanization Assay and PO Activity

For the melanization assay, twenty *O. formosanus* workers were placed into culture dishes (9 cm) with moist sterilized filter paper. One microliter of 8.75 × 10^10^ CFU/mL SM1 or 2.14 × 10^10^ CFU/mL Bt was placed on each worker’s pronotum (treatment group) (*n* = 20 workers per replicate) using an injection needle (PB600-1 Repeating Dispenser, Hamilton Co., Reno, NV, USA). For controls, one microliter of culture medium was placed on each worker’s pronotum (*n* = 20 workers per replicate). The experiment was replicated three times independently. The SM1 and Bt concentrations were predetermined prior to the experiments using dilution coating method. The hemolymph of *O. formosanus* workers was collected on ice at 24 h post infection (p.i.). The hemolymph and 100 µL PBS were mixed in Eppendorf tubes, then incubated at 28 °C for 4 h and then observed the qualitative level of melanization.

For PO activity assay, the termites of the 3 h treatment groups were treated by one microliter of 8.75 × 10^10^ CFU/mL SM1 or 2.14 × 10^10^ CFU/mL Bt for 3 h (*n* = 20 workers per replicate), while the termites of the 3 h control groups were treated by one microliter of culture medium for 3 h (*n* = 20 workers per replicate). In addition, the 6 h, 12 h or 24 h treatment groups and corresponding controls were collected in the same way. The experiment was replicated three times independently. Each sample was then homogenized manually on ice with 500 µL phosphate buffer solution (PBS, 0.1 M, pH = 7.5) using a plastic grinding pestle (TIANGEN, Beijing, China). Each homogenate was centrifuged by a 5471R centrifuge (Eppendorf, Hamburg, Germany) at 5900 g for 5 min at 4 °C, and then the supernatant was transferred into a new Eppendorf tube and centrifuged at 13,300× g for 30 min at 4 °C. The final supernatant was used to test PO activity. According to a previous study [56], bovine serum albumin (BSA) was set as a standard for determining the protein content of the supernatant, and the absorbance was detected at 595 nm in a Model 680 microplate reader (Bio-Rad, Hercules, CA, USA). PO activity was measured according to a previous study [57]. This mixture contained 30 µL of the supernatant, 135 µL PBS, and 135 µL of 0.0375 moL catechol. The absorbance value was detected at 420 nm for 2 min with a Model 680 microplate reader. A mixture that contained only catechol (CK) and PBS was used as a control and measured. The experiment was independently repeated three times. The results are indicated as the absorbance value of enzyme activity per mg protein (ΔOD420 nm·min^−1^·mg protein^−1^).

### 4.3. Cloning of the Full-Length Prophenoloxidase Gene of O. formosanus

Total RNA from non-treated *O. formosanus* workers was extracted using TsingZol Total RNA Extraction Reagent (TSP401, Tsingke Biotechnology Beijing, Co., Ltd., Beijing, China) according to the manufacturer’s protocol. The integrity and quantity of RNA were checked using 1% agarose gel electrophoresis and a NanoDrop spectrophotometer (Thermo Fisher Scientific, Waltham, MA, USA). *O. formosanus* cDNA was synthesized using an Evo M-MLV Plus 1st Strand cDNA Synthesis Kit (AG11615, Accurate Biotechnology, Hunan, Co., Ltd., Changsha, China) according to the manufacturer’s protocol, and cDNA was used as template to clone *OfPPO* gene. The PCR primers specific to *OfPPO* were designed using Premier 5.0 based on the full-length sequence of *O. formosanus* PPO obtained from the full-length transcriptome data (Table 2). PCR was executed using PrimeSTAR Max DNA Polymerase (Takara, Dalian, Liaoning, China). The PCR program was set as follows: 98 °C for 3 min; 35 cycles of 98 °C for 10 s, 52 °C for 15 s and 72 °C for 60 s; and an extension cycle of 72 °C for 5 min. The *OfPPO* DNA was subcloned into the TA clone vector and sequenced by Sangon Biotech (Shanghai) Co., Ltd., Shanghai, China.

### 4.4. Characteristics of OfPPO and Phylogenetic Analysis

The amino acid sequences were deduced from the NCBI Open Reading Frame (ORF) finder (https://www.ncbi.nlm.nih.gov/orffinder/, accessed on 20 June 2022). Then, their molecular weight and isoelectric point were calculated by the Expert Protein Analysis System (EXPASY) proteomics server (http://www.expasy.org/, accessed on 20 June 2022). The amino acid sequences of *OfPPO* were aligned with several known characterized PPOs from *C. formosanus*, *Z. nevadensis*, *B. germanica*, *B. mori*, *P. xylostella*, *S. frugiperda*, *B. dorsalis*, *T. castaneum* and *T. madens* to analyze conserved OfPPO motifs using DNAMAN multiple sequence alignment analysis (Version 6, Lynnon Biosoft Co., San Ramon, CA, USA). To construct the phylogenetic tree and explore the relationship between OfPPO and other PPOs, we chose prophenoloxidase as a keyword to search the nonredundant database from the NCBI website (https://www.ncbi.nlm.nih.gov/, accessed on 20 June 2022). Multiple amino acid sequence alignment analysis was performed using MEGA X (version 10.1) and Clustal X software (version 2.1). The phylogenetic tree was constructed using the neighbor-joining (NJ) method in MEGA X with 1000 bootstrap replicates.

### 4.5. OfPPO Expression in O. formosanus Using qPCR

Total RNA was extracted from each sample using TsingZol Total RNA Extraction Reagent, and mRNA was first reverse transcribed to cDNA using PrimeScript™ RT Master Mix (Takara, Dalian, Liaoning, China) according to the manufacturer’s protocol and subsequently verified by qPCR using 2×TSINGKE Master qPCR Mix (TSE201). In general, qPCR was carried out in a 20 µL volume consisting of 1 µL of cDNA (100 ng/uL) and 10 µL of 2×TSINGKE Master qPCR Mix, 0.4 µL of ROX Reference Dye II, 0.8 µL each of 10 µM forward and reverse primer and 7 µL of ddH_2_O using ViATM 7 Real-time PCR system (Applied Biosystems, Foster City, CA, USA). The qPCR program was 95 °C for 60 s, followed by 40 cycles of 95 °C for 10 s and 60 °C for 30 s, after which the melting curve was analyzed. Each primer for qPCR was designed using Premier 5.0 software according to the gene sequence. *GAPDH* and *RPS18* were used as internal reference genes. All primers are listed in Table 2. The amplification efficiency of primers was tested using Linreg PCR software (Version: 2016.1), according to the method described by Ramakers [58]. All primer sets utilized in this study had mean amplification efficiency values ranged between 1.7 and 2.0. Caste- and tissue-specific expression experiments were calculated using the 2^−∆Ct^ method, and other experiments were calculated using the 2^−∆∆Ct^ method [59,60]. Three replicates were performed for each treatment independently.

### 4.6. In Vitro Synthesis of dsOfPPOs (ds*OfPPO*1 and ds*OfPPO*2)

Two ds*OfPPO* sequences were designed based on the copper-binding site B motif and C-terminal conserved motif to evaluate the RNAi efficiency. Two ds*OfPPO*s were amplified from the *OfPPO* subcloned vector using ds*OfPPO*1 and ds*OfPPO*2 primers, and the restriction enzyme cutting sites were added to each primer. The PCR products of ds*OfPPO*s were digested by restriction enzyme, purified using a DNA Gel Extraction Kit (TSP601-50, Tsingke Biotechnology Co., Ltd., Beijing, China), and cloned into vector L4440 to produce ds*OfPPO*s.

The recombinant plasmids of ds*OfPPO*1 and ds*OfPPO*2 were transformed into *E. coli* HT115 competent cells [61,62]. The transformed bacteria were cultured in LB medium containing 100 μg/mL ampicillin and 12.5 μg/mL tetracycline at 37 ℃ for 12 h, with continuous shaking (200 rpm). A 50 μL aliquot of cultured bacteria was added to 50 mL of fresh LB medium containing 100 μg/mL ampicillin and 12.5 μg/mL tetracycline and allowed to grow until OD_600_ = 0.6–0.7. In order to induce the synthesis of dsRNA, isopropyl-beta-D-thiogalactopyranoside (IPTG, final concentration of 0.8 mM) was added to the bacteria culture and the mixture was cultured continuously at 37 °C for 12 h. Fifty microliter of the resulting bacterial culture was utilized to isolate dsRNA using TsingZol Total RNA Extraction kit. The integrity and quantity of RNA were analyzed using 1% agarose gel electrophoresis and a NanoDrop spectrophotometer. The dsRNA isolates were used for subsequent RNAi experiments. Double stranded RNA of GFP was used as a control dsRNA treatment and an expression plasmid containing the GFP gene was utilized to produce ds*GFP* using the same procedures that generated ds*OfPPO*s.

### 4.7. RNAi Efficiency Evaluation after Treatment with dsRNA

For triplicate dsRNA treatment experiments, twenty *O. formosanus* workers in a culture dish covered with wet filter paper were fed with 0.4 mL solution containing 1 μg/μL ds*OfPPO* and 10% (*w*/*v*) Nile blue (to recognize whether *O. formosanus* workers fed the fluid). The positive control group was fed with 0.4 mL ds*GFP* and Nile blue, and the negative control group was fed with 0.4 mL sterilized water containing Nile blue. Each treatment was repeated three times. At 3, 6, 12, and 24 h post treatment, the workers whose intestines turned blue by visual observation were collected and subjected for qPCR evaluation for RNAi interference efficiency on *OfPPO* gene expression.

### 4.8. The Role of OfPPOs in Resisting SM1 and Bt Infection

A number of *O. formosanus* workers for this experiment were placed into culture dishes with 0.4 mL of sterilized water and Nile blue (negative control), ds*GFP* and Nile blue (positive control), ds*OfPPO*s (ds*OfPPO*1 or ds*OfPPO*2) and Nile blue (treatment group) for 6 h. Then, workers from the negative control, positive control and treatment groups were divided into two groups (uninfected and bacterial infection groups), respectively. One microliter of culture medium was placed on each worker’s pronotum (control group), and 1 μL of SM1 (8.75 × 10^10^ CFU/mL) or 1 μL of Bt (2.14 × 10^10^ CFU/mL) was placed on each worker’s pronotum (treatment group). Each treatment contained 20 workers and was run in triplicates. The experiments were monitored hourly until one of the following conditions was met: (1) all members in an infected group died, and the mortality rate in the uninfected group was less than 20%. (2) The mortality rate in the uninfected group was at least 20% regardless of the mortality rate in the infected group.

### 4.9. Statistical Analysis

InStat software version 3.05 (GraphPad, San Diego, CA, USA) was used to analyze the data. The significant differences between the two samples in the PO activity and *OfPPO* expression assays were analyzed using a *t* test. One-way analysis of variance and Tukey’s multiple comparisons were used to analyze caste- and tissue-specific gene expression and RNA interference assays. A *p* value < 0.05 indicated a significant difference. For the survival assay, GraphPad Prism version 8.0.2 (GraphPad Software, San Diego, CA, USA) was used to analyze the data according to the log-rank (Mantel–Cox) test.

## Figures and Tables

**Table 1:** Percentage of similarity among six PPO amino acid sequences calculated using MEGA X.

Name	OfPPO	CfPO2	ZnPO2	BgPO1	CsPO1	TcPPO2
OfPPO	100.0	92.80	87.90	79.34	60.84	63.40
CfPO2		100.0	89.05	80.56	60.12	62.96
ZnPO2			100.0	80.56	59.68	61.93
BgPO1				100.0	59.82	63.70
CsPO1					100	76.83
TcPPO2						100

Of: *Odontotermes formosanus*; Cf: *Coptotermes formosanus*; Zn: *Zootermopsis nevadensis*; Bg: *Blattella germanica*; Cs: *Coccinella septempunctata*; Tc: *Tribolium castaneum*.

**Table 2:** Primers used for this study.

Primer Names	Forward Primer Sequences (5′-3′)	Reverse Primer Sequences (5′-3′)	Annealing Temperature	Experiments
*OfPPO*	ATGTCAAGACCAGCACAATC	TCATGAGAGCCGGTTTG	52 °C	Full-length amplification
ds*OfPPO*1	CGAGCTCGGTACAACTTCGAACGGCTG	TCCCCCGGGGCTTGCCTCCATCAGGTTAC	57 °C	RNAi
ds*OfPPO*2	CGAGCTCTCCAACAACGATGATGATAAGATA	TCCCCCGGGGTTTGGCTCATCTCTACCCTG	57 °C	
ds*GFP*	ATCGGAGCTCTAGTTGAACGGATCCATCTTCA	CCCAAGCTTAGAACTTTTCACTGGA	54 °C	
*OfPPO*	GTTGATCTGTCCCGAGGTATTG	GCTGTTCTCAGCCTGAATCTTA	60 °C	qPCR
*GAPDH*	TCGTATTGGCCGTCTTGTGC	AGCGACCATGGGTGGAATCAT	60 °C	
*RPS18*	ATGGCAAACCCCCGTCAGTA	CATACCACGATGCGCACGAA	60 °C	

**Note:** The underline within primers indicated the restriction sites; *GAPDH* and *RPS18* were the internal reference genes in this study.

## Data Availability

Data are contained within the article and supplementary material.

## Supplementary Materials

The following supporting information can be downloaded at: https://www.mdpi.com/article/10.3390/ijms24010406/s1.
