# Supplementary material for: Prophenoloxidase of Odontotermes formosanus (Shiraki) (Blattodea: Termitidae) Is a Key Gene in Melanization and Has a Defensive Role during Bacterial Infection
Source: Int J Mol Sci. 2022 Dec 26;24(1):406. doi: 10.3390/ijms24010406 (PMC9820534; doi:10.3390/ijms24010406)
Supplement: Supplementary file 1 [file ijms-24-00406-s001.zip › ijms-1965326-supplementary.pdf]

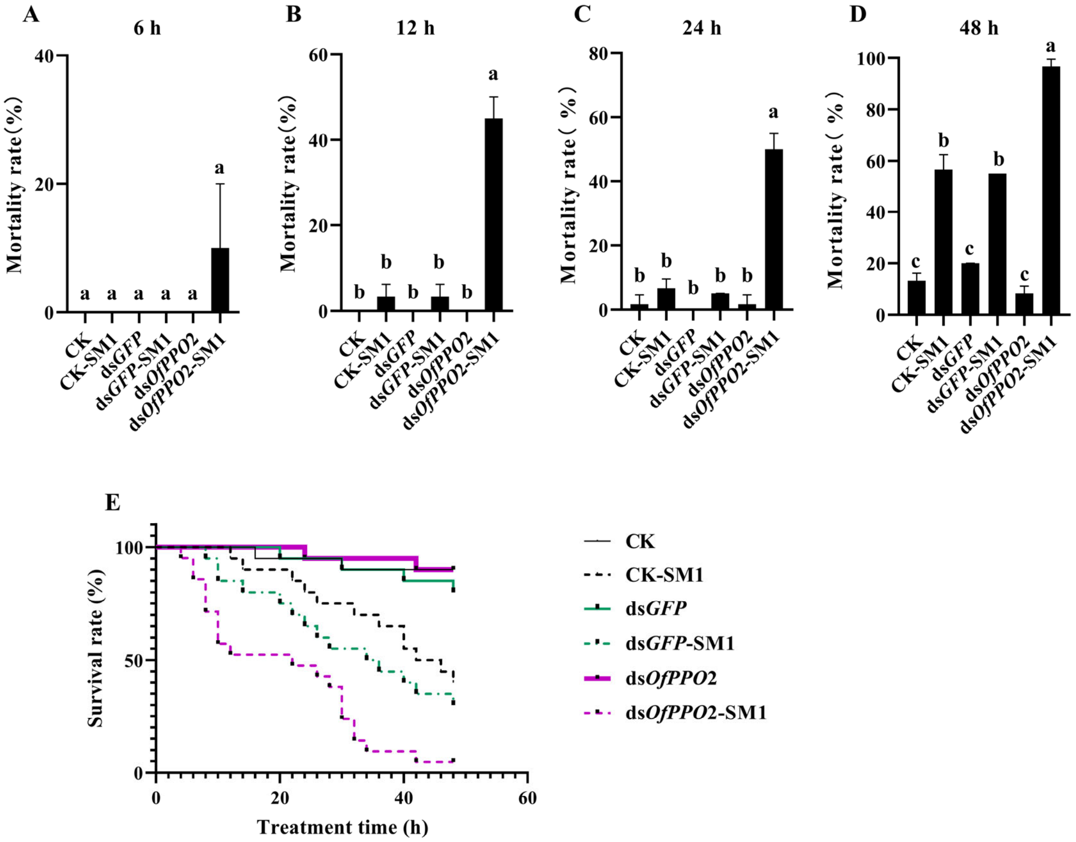

Figure S1: Bioassay test of SM1-challenged *O. formosanus* treated with ds*OfPPO*2. (A) The mortality rate of *O. formosanus* in each group at 6 h. (B) The mortality rate of *O. formosanus* in each group at 12 h. (C) The mortality rate of *O. formosanus* in each group at 24 h. (D) The mortality rate of *O. formosanus* in each group at 48 h. (E) The survival rate of SM1-challenged *O. formosanus* treated with ds*OfPPO*2. The data are presented as the mean ± SD of three replicates. In these figures, the same letters indicate no significant differences in the mortality of *O. formosanus* ($p > 0.05$).

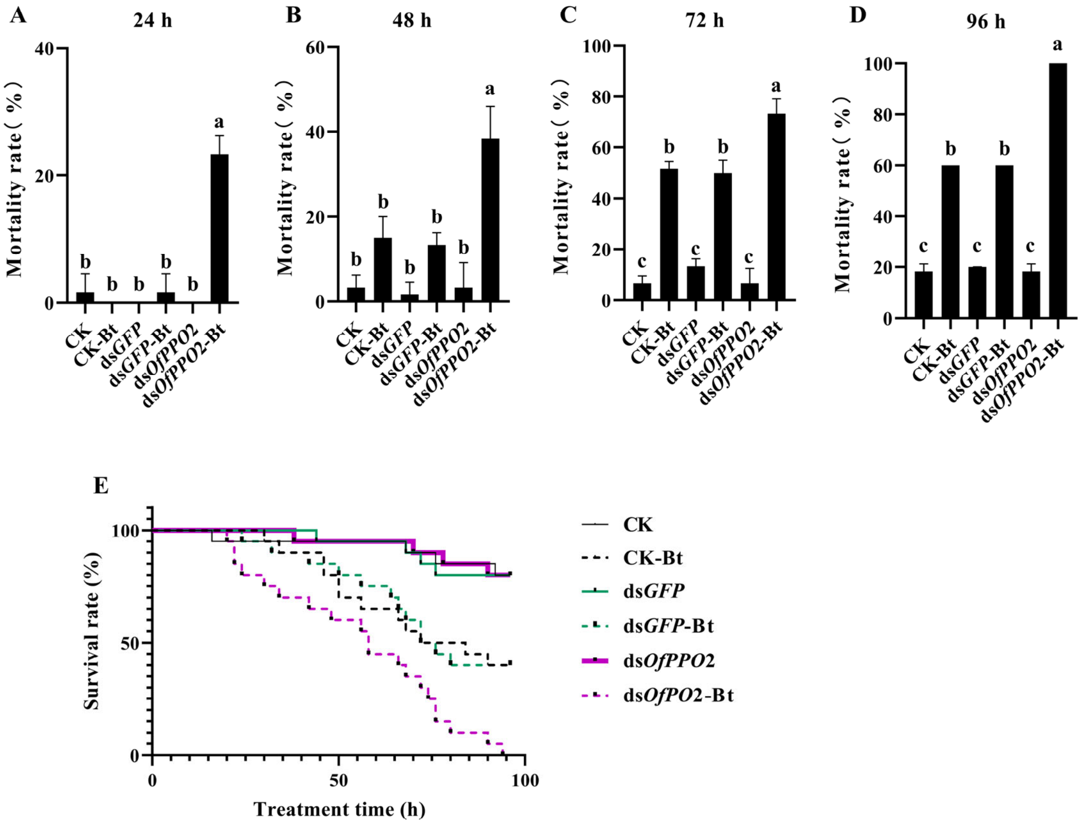

Figure S2: Bioassay test of Bt-challenged *O. formosanus* treated with ds*OfPPO*2. (A) The mortality rate of *O. formosanus* in each group at 24 h. (B) The mortality rate of *O. formosanus* in each group at 48 h. (C) The mortality rate of *O. formosanus* in each group at 72 h. (D) The mortality rate of *O. formosanus* in each group at 96 h. (E) The survival rate of Bt-challenged *O. formosanus* treated with ds*OfPPO*2. The data are presented as the mean ± SD of three replicates. In these figures, the same letters indicate no significant differences in the mortality of *O. formosanus* ($p > 0.05$).
